# Clinical and Basic Studies on Therapeutic Efficacy of Herbal Medicines against Mycobacterial Infections

**DOI:** 10.3390/medicines6020067

**Published:** 2019-06-19

**Authors:** Haruaki Tomioka, Yutaka Tatano, Toshiaki Shimizu, Chiaki Sano

**Affiliations:** 1 Department of Basic Medical Science for Nursing, Department of Primary Education, Yasuda Women’s University, Hiroshima 731-0153, Japan; 2 Department of Microbiology and Immunology, Shimane University School of Medicine, Izumo 693-8501, Japan; 3 Department of Pharmaceutical Sciences, International University of Health and Welfare, Otawara 324-8501, Japan; tatanoy@iuhw.ac.jp; 4 Department of Nutritional Sciences, Yasuda Women’s University, Hiroshima 731-0153, Japan; shimizu-t@yasuda-u.ac.jp; 5 Department of Community Medicine Management, Shimane University School of Medicine, Izumo 693-8501, Japan; sanochi@med.shimane-u.ac.jp

**Keywords:** herbal medicines, medicinal plants, immunoadjunctive agents, host-directed therapeutics, mycobacterial infections

## Abstract

The high incidence of tuberculosis (TB) in developing countries, the resurgence of TB in industrialized countries, and the worldwide increase in the prevalence of *Mycobacterium avium* complex infections are important global health concerns. However, the development of novel antimycobacterial drugs is currently making very slow progress. Therefore, it is considered that devising improved administration protocols for clinical treatment against intractable mycobacteriosis using existing chemotherapeutics is more practical than awaiting the development of new antimycobacterial drugs. The regulation of host immune responses using immunoadjunctive agents may increase the efficacy of antimicrobial treatment against mycobacteriosis. In particular, the mild and long-term up-regulation of host immune reactions against mycobacterial pathogens using herbal medicines may be beneficial for such immunoadjunctive therapy. This review focuses on the current status regarding basic and clinical studies on protocols using herbal medicines, including medicinal plants, useful for the clinical treatment of intractable mycobacterial infections.

## 1. Introduction

Tuberculosis (TB), the leading cause of infection-related death in the world, and the global increase in the prevalence of non-tuberculous mycobacterial infections in immunocompromised hosts, especially acquired immunodeficiency syndrome (AIDS) patients, are serious international health concerns. Therefore, the development of potent new antituberculosis drugs is urgently needed [1,2]. Indeed, the treatment of pulmonary *Mycobacterium avium* complex (MAC) infections is still difficult even with the use of multi-drug regimens consisting of new macrolides (clarithromycin and azithromycin) and new rifamycins such as rifabutin [3,4,5]. Although limited numbers of new drugs, including delamanid (nitro-dihydro-imidazooxazole) and bedaquiline (diarylquinoline), have been approved for clinical use for the treatment of TB patients as second-line drugs, it may take a long time to achieve the development of more favorable chemotherapeutics for the clinical treatment of intractable mycobacterioses. Thus, the strategy to improve the therapeutic efficacy of existing antimycobacterial drugs by the aid of combining the use of immunoadjunctive drugs may be more practical than awaiting the development of new antimycobacterial drugs in the future [6,7]. 

However, the clinical use of immunoadjunctive agents in combination with antimycobacterial chemotherapy is still associated with certain serious problems and dilemmas, such as the high cost and occasionally strong side effects. In addition, most immunoadjunctive drugs usually display only modest efficacy in potentiating host defense mechanisms against mycobacteria, partly due to the induction of macrophage-deactivating cytokines and prostaglandin E_2_ during the course of the long-term administration of immunopotentiating agents [6,8]. Thus, for immunoadjunctive therapy against mycobacterial infections, it may be favorable to use agents that mildly and steadily up-regulate the cell-mediated immunity of patients with mycobacteriosis during the course of long-term therapy, without inducing excess immune responses that may cause immune deviation leading to the generation of macrophage-deactivating cytokines. Herbal medicines, especially traditional Chinese herbal medicines (CHMs), are suitable for this purpose, since these drugs generally potentiate host immunity in a mild fashion and they can be prescribed for patients at relatively low cost. This review deals with the current status and future prospects regarding the development of immunoadjunctive protocols for the clinical treatment of intractable mycobacterial infections using various herbal medicines, including medicinal plants.

## 2. General Aspect of Immunoadjunctive Therapy for the Clinical Treatment of Mycobacteriosis

Host-directed therapeutics (HDTs) show adjunctive effects against mycobacterial infections through the inhibition of host-related factors, as follows: (1) factors required for the expression of bacterial pathogenesis, (2) factors related to the potentiation of innate and acquired immunity against mycobacterial pathogens, (3) factors acting in the reduction of the host’s responses related to the progression and exacerbation of mycobacteriosis, (4) factors neccessary for the recovery of immune responses that are more or less suppressed during the advanced stages of mycobacterial infections, and (5) factors causing the prevention of lung injury due to the overexpression of antimycobacterial immunity by modifying specific mechanisms causing lung inflammation and tissue damage [7,9,10,11]. 

Therefore, it is possible to devise regimens to treat patients with intractable mycobacteriosis using conventional antimycobacterial drugs in combination with immunomodulators. For this purpose, Th-1 cytokines (IFN-γ, IL-2) and Th-1 response-inducing cytokines (IL-12, IL-18) have been studied for their immunoadjunctive activity in chemotherapy against mycobacterial infections [6]. In addition, some promising HDTs, including vitamin D, non-steroidal anti-inflammatory drugs, autophagy inducers, galactosylceramide, poloxamer, picolinic acid, and heat-inactivated *Mycobacterium vaccae*, are currently being developed for chemotherapy against refractory mycobacteriosis [6,7,9,10]. However, adjunctive immunotherapy using these HDTs is still associated with serious problems and dilemmas, as previously described in the Introduction section. 

Thus, it is important to screen for new drugs with mild immunopotentiating effects, which do not induce immunosuppressive cytokines, including TGF-β, IL-10, and IL-13, during administration for long periods of time. Such kinds of immunomodulatory agents are expected to make it possible to establish new regimens of adjunctive immunotherapy highly effective against mycobaceteriosis. Some kinds of herbal medicines/medicinal plants may be appropriate for this purpose, as described below. 

## 3. Immunological Effects of Herbal Medicines/Medicinal Plants, and Their Chemical Components in Modulating Host Antimycobacterial Resistance

As described below, certain types of herbal medicines/medicinal plants, including Niubeixiaohe, maobushisaishinto, yokuinin, and a water extract of *Ranuncli ternati*, *Sophorae flavescentis*, *Prunella vulgaris*, and *Stellera chamaejasme* have been demonstrated to exhibit therapeutic effects against mycobacterial infections induced in experimental animals, partly through their immunoadjunctive effects causing the potentiation of Th1 cell-mediated cellular immunity of hosts against mycobacterial pathogens [12,13,14]. Similarly, certain herbs and their chemical components also exhibit an action to modulate host antimicrobial cellular immunity in cases of microbial infections due to pathogens other than mycobacteria. Moreover, some kinds of chemical components of herbal medicines/medicinal plants exhibit an immuno-regulatory/ immunosuppressive action possibly due to the induction of a regulatory T cell (Treg cell) subset and M2-type macrophages (Table 1).

### 3.1. Curcumin

It has been demonstrated that some herbs and their chemical components elicit immunoregulatory responses in hosts due to immunodeviation, characterized by Th2 and Treg cell expansion and polarization. For instance, this has recently been reported by some researchers concerning the immunoadjunctive effects of curcumin in MTB-infected hosts as follows: curcumin is one of the major polyphenol components contained in the herb turmeric (*Curcuma longa*), and exhibits effects to reduce the disturbance of blood flow, sedation and analgesic effects, and a stomachic effect [27]. Notably, curcumin potentiated anti-*Mycobacterium tuberculosis* (MTB) antimicrobial activity of human macrophages, possibly through the up-regulation of apoptosis and autophagy mediated by the activation of caspase 3 [15].

Curcumin has also been demonstrated to exhibit appreciable blocking effects against daunorubicin-induced nephrotoxicity in rats [16]. In this case, curcumin blocked the expansion of M1 macrophages induced with daunorubicin accompanied by an increase in the expressions of TNF-α, IL-6, CD86, and COX2 in host macrophages through the activation of ERK1/2 and NF-κB-mediated signaling pathways. In this context, M1 and M2 macrophage populations are known to have distinct phenotypes because of their differential profiles of gene expression [28,29,30]. M1 macrophages participate as inducer and effector cells in polarized Th1 responses and play roles in resistance against bacterial pathogens and tumors [28,29]. Typical M1 macrophages possess a phenotype with the high-level production of IL-12 and IL-23 but low-level expression of IL-10. They are also efficient producers of cytotoxic effector molecules, such as reactive oxygen species and reactive nitrogen intermediates and proinflammatory cytokines, including IL-1β, TNF-α, and IL-6. 

On the other hand, M2 macrophages have a phenotype with the low-level production of IL-12 and IL-23 but high-level expression of IL-10. In general, M2 macrophages are characterized by the low-level production of proinflammatory cytokines. Indeed, M2 macrophages play important roles in polarized Th2 reactions: (1) they promote the killing and encapsulation of helminth parasites; (2) they promote tumor progression and tissue repair and remodeling; and (3) they exert immunoregulatory and anti-inflammatory actions [28,29]. In this context, it has been reported that curcumin inhibited MTB’s 19-kD lipoprotein-mediated TLR2 activation of macrophages, thereby blocking the TLR2-mediated apoptosis of host macrophages [31]. Since TLR2 expression is characteristic of M1 macrophages, curcumin is considered to suppress the cellular functions of these macrophages. 

These findings may indicate that curcumin exhibits significant immunoregulatory effects in hosts. However, it should be noted that curcumin may be a deceptive molecule according to some chemists, as described in a recent commentary presented in the journal *Nature* [32]. The commentary indicates the possibility that curcumin may disrupt cell membranes, duping our attempts to search for new drugs based on assays that aim to identify drugs targeting specific cell-membrane proteins, and that curcumin may degrade into other compounds exhibiting different properties.

### 3.2. *Astragalus* Polysaccharide and Polyphenol

In the case of *Astragalus membranaceus*, which is traditionally used for treatment of infections of the mucous membranes, especially the urinary and respiratory tracts [33]. Its polysaccharide (*Astragalus* polysaccharide: APS) and saponin (a polyphenol component called “Astragaloside”) fractions have been demonstrated to potentiate the ability of macrophages to phagocytose MTB bacilli and up-regulate macrophage production of inflammatory cytokines including IL-6, IL-1β, and TNF-α [17]. This is consistent with previous findings whereby *Astragalus membranaceus* potentiated macrophage phagocytosis of *Candida albicans* through the activation of C3b and Fc receptors and up-regulated macrophage functions via the activation of TLR4 [18]. 

In this context, Hou et al. recently reported an interesting finding concerning the usefulness of APS in the clinical control of bacteremia, as follows [19]: the intraperitoneal injection of APS to mice reduced the progression of bacteremia induced by endogenous infection with intestinal bacterial flora. In this case, ASP up-regulated the expansion of Th1 and Th17 cells and the generation of IFN-γ, IL-2, and IL-17, while it down-regulated the induction of Treg and Th2 cells concomitant with the reduction of IL-4 generation. These findings support the hypothesis that ASP causes the up-regulation of host cellular immunity against pathogenic microorganisms. 

### 3.3. Triptolide of *Tripterygium Regelii*

It was reported by Qiu et al. that triptolide, a diterpene triepoxide, of a medicinal plant called *Tripterygium regelii*, which is effective against inflammatory diseases including rheumatoid arthritis, systemic lupus erythematosus, and atopic dermatitis (atopic eczema) [34], suppressed IL-2 production by TCR-stimulated T cells by inhibiting the activation of some transcription factors, such as NF-κB and NF-AT [20]. Concerning the anti-inflammatory action of triptolide, Qiu et al. reported an interesting finding whereby this agent suppressed macrophage production of IL-12 and IL-23, responding to LPS stimulation through up-regulation of the expression of the gene encoding CCAAT/enhancer-binding protein-α (C/EBPα), which inhibits transcription of the IL-12 p40 gene by blocking its promoter activity [20].

### 3.4. Berberine of *Coptis Japonic* and *Phellodendron Amurense*

Qin et al. reported an interesting finding whereby berberine, an isoquinoline alkaloid of medicinal plants including *Coptis japonic* and *Phellodendron amurense* with anti-inflammatory activity [35], was efficacious in the amelioration of murine experimental autoimmune encephalomyelitis induced by myelin oligodendrocyte glycoprotein (MOG) [21]. In this case, berberine inhibited the expansion of Th1 and Th17 cells induced by MOG stimulation but not Treg cell expansion. This inhibitory effect of berberine was associated with its blocking action against the phosphorylation of JAK1, Tyk2, STAT1, STAT3 and STAT4, and RORγt expression. Notably, berberine was found to suppress the ability of CD11b^+^ antigen-presenting cells, leading to dysfunction of the recognition of MOG antigen by naive T cells [21]. Therefore, berberine is considered to exhibit suppressive effects against autoimmune diseases and allergy by reducing excess levels of immune reactions.

### 3.5. Artemisinin of *Artemisia Annua*

Artemisinin, a lactone component of a medicinal plant called sweet annie (*Artemisia annua*), possessing excellent antimalarial activity and immunomodulatory and angiogenetic effects [36], was reported by Zhao et al. to suppress the proliferative response of TCR-stimulated Th cells. Notably, artemisinin potentiated Treg expansion due to its blocking effect against the mTOR-mediated signaling pathway [37]. Indeed, the inhibition of mTOR activity with rapamycin caused the blocking of Akt protein-mediated inhibition of arteminisin’s action in expanding Treg cells. These findings indicate that artemisinin suppresses excess levels of cellular immunity, which is mediated by Th1 cell subsets, through the artemisinin-mediated induction of Treg cells.

### 3.6. Andrographolide of *Andrographis Paniculata*

A recent study by Liao et al. clarified interesting activity of a medicinal herb called Senshinren (*Andrographis paniculata*) in the treatment of steroid-resistant airway hyper-responsiveness in patients with asthma. LPS and IFN-γ are known to up-regulate the murine macrophage function to generate IL-27 [22]. Differing from macrophages stimulated with LPS or IFN-γ singly, macrophages stimulated with LPS in combination with IFN-γ (LPS/IFN-γ-stimulated macrophages) show resistance to the inhibitory action of dexamethasone against macrophage IL-27 expression. According to the authors, andrographolide, a diterpenelactone component of Senshinren, restored the lowered sensitivity of LPS/IFN-γ-stimulated macrophages to dexamethasone by blocking the activation of PI3K/Akt-, NF-κB-, and p38 MAPK-mediated signaling pathways that are crucial in the establishment of the steroid-resistance of LPS/IFN-γ-stimulated macrophages. Moreover, in vivo experiments by Liao et al. indicated the following: dexamethasone alone failed to inhibit LPS/IFN-γ-induced IL-27 production and airway hyper-responsiveness in mice [22]. However, andrographolide significantly restored the suppressive effect of dexamethasone on LPS/IFN-γ-induced IL-27 production in lung macrophages, and andrographolide was actually efficacious in treating steroid-resistant airway hyper-responsiveness in mice. These findings are considered helpful for the development of new regimens using CHMs useful for the clinical control of intractable asthma.

### 3.7. Piperlongumine of *Piper Longum*

It was recently reported that piperlongumine, an alkaloid component of a herb called Hihatsu (*Piper longum*), which is generally known to exhibit antimicrobial effects, a vasodilatory action, inhibitory effects against arteriosclerosis plaque formation and platelet aggregation, and an analgesic action [38], was effective for the clinical treatment of and prophylaxis against rheumatoid diseases [23]. In this study, it was found that piperlongumine inhibited the maturation of murine bone marrow-derived dendritic cells responding to LPS stimulation, causing the reduction of CD80/CD86 expression, and the manifestation of this effect by piperlongumine was accompanied by blocking of the up-regulation of inflammatory cytokines (such as IL-12, IL-6, and TNF-α) due to the activation of dendritic cells. Interestingly, this phenomenon was also accompanied by the piperlongumine-mediated inhibition of the production of reactive oxygen species by LPS-stimulated dendritic cells. Notably, LPS stimulation is known to up-regulate reactive oxygen species generation by dendritic cells through the activation of signal transduction pathways mediated by PI3K/Akt and p38 and JNK MAP kinases (MAPKs). Indeed, Xiao’s study also indicated that piperlongumine actually inhibits these signal transduction axes. These in vitro phenomena were also confirmed by in vivo experiments using a mouse experimental model for collagen-induced arthritis [23]. These findings strongly indicate that Hihatsu and its alkaloid component piperlongumine exert favorable therapeutic effects against rheumatoid diseases.

### 3.8. Osthole of *Cnidium Monnieri*

Osthole, 7-methoxy-8-(3-methyl-2-butenyl) coumarin, is a pure compound isolated from the seeds of *Cnidium monnieri*, has received considerable attention because of its favorable pharmacological properties, including anti-inflammatory, immunomodulatory, antiallergic, anticancer, antihepatitis, neuroprotective, and osteogenic effects [39]. Chiang et al. demonstrated that the oral administration of osthole to asthmatic mice suppressed Th2 cell-mediated allergic asthma by inhibiting the production of Th2-type cytokines including IL-4, IL-5, and IL-13 but not IL-10, thereby causing the reduction of immunoglobulin E generation [24]. Chiang et al. also noted the reduction of regulatory T-cell induction in osthole-treated asthmatic mice [24]. The in vitro analyses revealed that osthole-treated bone-marrow-derived dendritic cells were partially mature, showing a phenotype that secreted a high level of IL-10, while proinflammatory cytokines, including IL-12, IL-6, and TNF-α were produced at low levels. These partially mature dendritic cells exhibited an immunosuppressive action by suppressing effector T-cell functions or inducing Treg cells. 

### 3.9. *Glycyrrhiza Uralensis*

The root of *Glycyrrhiza uralensis* has been used as a herbal medicine in the world for a long period. *G. uralensis* is a natural sweetener and used in treating several diseases, including diabetes, lung diseases, and coughs [40]. Yang et al. demonstrated that this herbal medicine inhibited the production of caspase-1 resulting in reduction of mature IL-1β generation induced by NLRP3 inflammasome activation in macrophages [25]. Furthermore, *G. uralensis* markedly inhibited diet-induced adipose tissue inflammation and IL-1β and caspase-1 production in white adipose tissue. The results indicate that *G. uralensis* may be useful for the treatment of patients with NLRP3 inflammasome-associated inflammatory diseases.

### 3.10. Gastrodiae Rhizoma

Gastrodiae Rhizoma (GR), dried rhizome of *Gastrodia elata*, is a traditional herbal medicine in western Asia, which is used for the treatment of headaches, hypertension, oxidative stress, mental disorders, and inflammation [41]. Recently, Park et al. demonstrated that GR fermented with *Lactobacillus brevis* at 37°C for a few days reduced macrophage production of reactive oxygen species and prostaglandin E_2_ through activation of NF-κB-mediated signal pathways [26]. This suggests that the GR preparation may be efficacious for down-regulating inflammatory reactions in vivo.

### 3.11. Some Remarks on the Immunological Action of Chemical Components of Herbal Medicines

As described in this section, the immunoadjunctive effects of herbal medicines, including medicinal plants, can be divided into the following two actions: firstly, some kinds of herbal medicines/medicinal plants up-regulate the cellular immunity of hosts via the expansion and activation of M1 macrophages and Th1 lymphocytes. Secondly, other types of herbal medicines/medicinal plants down-regulate cellular immunity and inflammation in hosts via the differentiation and activation of M2 macrophages and Th2 and Treg lymphocytes.

In this section, we have mainly described recent findings regarding the immunological and molecular biological effects of chemical components, which are contained in some kinds of medicinal herbs. In this context, it should be noted that numerous plants serve as important sources of chemical entities supporting drug discovery. The rich traditions of herbal medicines/medicinal plants developed by a myriad of trials on human subjects over thousands of years present us with invaluable biomedical information just waiting to be uncovered using modern scientific approaches [42]. Further detailed studies are needed to elucidate the chemical and biological properties of active constituents of various types of herbal medicines/medicinal plants, which are promising as immunoadjunctive agents in the treatment of bacterial infections including mycobacteriosis.

## 4. In Vivo Efficacy of Herbal Medicines as Immunoadjunctive Therapeutics against Mycobacteriosis

### 4.1. Therapeutic Effects of Herbal Medicines against Experimental Mycobacterial Infections Induced in Mice and Rats

As described above, certain herbal mecicines/medicinal plants and their chemical components are promising as immunoadjunctive agents for clinical therapy against mycobacteriosis. In this section, we will discuss this on the basis of findings obtained by recent basic studies using experimental mycobacterial infections induced in mice and rats.

Firstly, Lu et al. reported that the mixed water extracts obtained from four herbs: *Ranuncli ternati* with detumescence effect, *Sophorae flavescentis* with anti-inflammatory, diuretic, and antipruritic effects, *Prunella vulgaris* with diuretic and antiphlogistic effects, and *Stellera chamaejasme* with antitumor activity, potentiated host resistance to MTB infection induced in rats [14]. This protective effect was largely due to the deviation toward Th1-type immune responses, characterized by the up-regulation of IFN-γ and IL-12 expressions concomitant with the down-regulation of IL-4 and IL-10 expressions in MTB-infected rats. 

Secondly, Liang et al. examined the therapeutic activity of the traditional Chinese herbal medicine Niubeixiaohe, consisting of seven herbs including *Fritillaria* with a moisture metabolism-improving effect, *Houttuynia* with diuretic effect, *Bletilla* with a hemostatic effect, and *Platycodon grandiflorus* with an expectorant effect, against MTB infection induced in mice [43]. Notably, this herbal medicine is widely used for the treatment of TB patients in China. In the study by Liang et al., water and ethanol extracts of Niubeixiaohe mildly inhibited bacterial growth in the spleen, causing a 0.4 to 0.7-log unit decrease in bacterial loads, but not in the lungs [43]. This effect was accompanied by the moderate improvement of histopathological features in the visceral organs of infected mice. Thus, the Niubeixiaohe regimen is considered to be modestly useful for TB therapy, although its efficacy is much weaker than that of chemotherapy based on the administration of antituberculous drugs.

Thirdly, Yifei Tongluo is a traditional Chinese medicine formulation containing multiple herbs, such as *Polygonatum sibiricum*, *Bletilla striata*, *Pseudostellariae radix*, *Stemona sessilifolia*, and *Ardisia japonica*, and has been widely used to maintain immune homeostasis. Treatment with Yifei Tongluo in combination with anti-tuberculous drugs has been reported to improve the clinical symptoms of patients with MDR-TB in China [44]. Using *Mycobacterium bovis* BCG-infected mice, Fan et al. demonstrated that the lung bacterial load in Yifei Tongluo-treated mice was significantly reduced as compared with untreated control mice [45]. This phenomenon was accompanied by alleviated pulmonary inflammation with a reduction of pro-inflammatory cytokine (IL-1β, TNF-α, and IL-6) levels and an increase of prostaglandin E_2_ levels in the blood. Notably, Th1 cells were significantly higher in the lungs of Yifei Tongluo-treated mice at early infection time (within two weeks after infection), suggesting that Yifei Tongluo-treatment down-regulates pulmonary inflammation, and consequently facilitates rapid Th1 cell infiltration into the lungs. However, the Th1 cells in the lungs were resolved faster at a later time (four weeks after infection) with a concomittant increase in regulatory T cells. Reduction of the mycobacterial burden associated with an improved tissue pathology, faster Th1 cell trafficking, and accelerated resolution of Th1 cells in the lungs of Yifei Tongluo-treated mice indicate that this herbal formulation may improve mycobacterial clearance by maintaining lung homeostasis and regulating T cells in the lung parenchyma.

Fourthly, Shimizu et al. reported the therapeutic efficacy of the herbal medicine maobushisaishinto (MBST) against MAC infection induced in mice [12]. MBST, a mixture of extracts from three medicinal herbs: *Ephedra*, *Aconitum*, and *Asarum*, has long been used in Japan to treat patients with the common cold. MBST contains a number of pharmacologically active components, including *Ephedra*-derived l-ephedrine and ephedran; *Aconitum*-derived aconitine, coryneine, and mesaconitine; and *Asarum*-derived methyleugenol, elemicin, l-asarinin, and higenamine. This herbal medicine is also efficacious in controlling perennial nasal allergy [46]. Indeed, it has been demonstrated to suppress passive cutaneous anaphylaxis induced experimentally in rats, possibly by inhibiting histamine release from mast cells. 

Shimizu et al. demonstrated that the therapeutic efficacy of the rifamycin derivative rifalazil against MAC infection induced in mice, as measured by the inhibition of MAC growth in the lungs, was moderately increased when it was given in combination with MBST, although MBST alone did not exhibit significant therapeutic effects [12]. Shimizu et al. demonstrated the following findings, particularly on the immunological mechanisms of the MBST-medicated potentiation of the therapeutic efficacy of rifalazil [12]: firstly, MBST treatment of macrophages promoted the rifalazil-mediated killing of intramacrophage MAC. However, MBST-treated macrophages showed decreased production of reactive nitrogen intermediates and reactive oxygen species, suggesting that these antimicrobial radicals are not decisive in determining the expression of macrophage anti-MAC activity. Secondly, the MBST-mediated potentiation of the in vitro and in vivo activities of rifalazil against MAC may be due in part to a reduction of macrophage IL-10 production by MBST. 

Indeed, IL-10 is known to down-regulate the anti-MAC antimicrobial activity of host macrophages [6]. Similar effects were also found for yokuinin, seeds of *Coix lacryma-jobi* var. frumentacea, having suppressive effects against macrophage IL-10 production [13]. This herbal medicine was mildly efficacious in inhibiting bacterial growth in MAC-infected macrophages [13]. In this context, it is noteworthy that 30 test herbal medicines other than MBST and yokuinin, did not exhibit such immunostimulatory effects characteristic of the two agents [47]. In any case, these findings support the possible usefulness of certain herbal medicines, such as Niubeixiaohe, MBST, and yokuinin, as adjunctive agents for the clinical treatment of intractable infections due to nontuberculous mycobacteria. 

### 4.2. Therapeutic Effects of Traditional Herbal Medicines against Clinical Control of Intractable Mycobacteriosis in Humans

Some herbal medicines have been reported to be mildly efficacious in the clinical treatment of patients with TB and non-tuberculous mycobacterial infections when administered alone or in combination with antimycobacterial drug regimens, as described below (Table 2). 

A recent study by Jiang et al. using meta-analysis to assess the efficacy of a herbal medicine-based therapeutic regimen as concomitant therapy against multidrug-resistant TB (MDR-TB) revealed the following [48]: meta analysis comprising 1,823 Chinese patients showed that Chinese herbal medicines given to TB patients with antimycobacterial chemotherapeutics resulted in a superior treatment success (Odds ratio = 1.33, *p* < 0.001) and radiological improvement (Odds ratio = 1.32, *p* < 0.001) with a low-level incidence of adverse effects of anti-TB drugs and similar rate of relapse as in the case of control patients given anti-TB chemotherapy alone. A similar finding was also reported by Wang et al. based on meta analysis comprising 3374 MDR-TB patients [49]. They noted the favorable effects of Chinese herbal medicines on the resorption rate of lung lesions, cavity closure rate, and relapse rate after chemotherapy.

Next, the *Qi*-boosting decoction of traditional Chinese medicines is thought to potentiate qigong (a holistic system of coordinated body posture and movement, breathing, and meditation used for the purposes of health, spirituality, and martial arts training) of humans, and is widely used to potentiate cardiopulmonary functions. The *Yin*-nourishing decoction is also widely employed to improve the nutritional status of patients (nourishment). It was recently reported that these regimens were effective in accelerating the negative conversion of TB bacilli in sputum and reducing lung lesions, when given to a patient with type 2 diabetes complicated with pulmonary TB in combination with chemotherapy using insulin and antituberculous drugs (isoniazid, rifampin, pyrazinamide, and ethambutol) [57].

The following are individual clinical cases showing the appreciable therapeutic efficacy of traditional herbal medicines, when used as immunoadjunctive agents for the clinical treatment of patients with mycobacterial infections. 

### 4.3. Clinical Studies on the Therapeutic Efficacies of Individual Herbal Medicines

#### 4.3.1. Ninjin’yoeito

Ninjin’yoeito (Ren Shen Yang Ying Tang) (12 herbs: including *Rehmannia glutinosa*, *Paeonia lactiflora*, *Daucus carota*, and *Astragalus membranaceus*) is known to improve the following symptoms: cough, chest pain, fever and night sweat, and anorexia. Inagaki et al. reported that ninjin’yoeito exhibited immunoadjunctive effects, when given to a patient with MAC infection in combination with antimycobacterial chemotherapy using clarithromycin and ciprofloxacin [51]. It was also found that the administration of ninjin’yoeito combined with anti-MAC chemotherapy was efficacious in accelerating the negative conversion of MAC bacilli in sputum, and improvement of the radiological features of the lungs of patients with MAC infection [51]. In addition, Nogami et al. indicated that 10-month monotherapy using ninjin’yoeito caused the negative conversion of bacterial loads in sputum counted by bacterial cultivation and microscopic observation in a patient with *M. fortuitum* infection. Notably, the chemotherapy using rifampin, clarithromycin, and ethambutol was not effective in improving symptoms of the patient [52]. It has also been reported that, when the alternatively prescribed “ninjin’yoeito” (11 herbs: including *Platycodon grandiflorus*, *Daucus carota*, and *Fritillaria verticillata*) was administered alone to a patient with *Mycobacterium xenopi* infection, the herbal medicine exhibited significant efficacy in increasing the patient’s body weight and, moreover, reducing sputum expectoration and cough [53]. 

#### 4.3.2. Hochuekkito

Hochuekkito (Bu Zhong Yi Qi Tang) (10 herbs including *Daucus carota*, *Astragalus membranaceus*, *Atractylodes lancea*, *Bupleurum falcatum*, and *Angelica acutilob*), is occasionally used in Japan for the control of a weak constitution, physical weariness, anorexia, and gastrointestinal hypomotility. A recent randomized controlled trial enrolling 18 patients with an advanced stages of MAC disease revealed the significant therapeutic efficacy of hochuekkito [50]. In this study, it was found that hochuekkito effectively blocked the increase in bacterial loads in the sputum, accompanied by radiological and nutritional improvements, during 24-week treatment in combination with anti-MAC antimicrobial chemotherapy [50].

#### 4.3.3. Saikanto

Saikanto (Chai Xian Tang) (9 herbs: including *Bupleurum falcatum*, *Coptis japonica*, *Pinellia*, *Daucus carota*, and *Zingiber officinale*) is known to be effective in reducing coughs and chest pain. It has also been reported that this traditional herbal medicine reduced inflammatory reactions and anemia of a patient with intractable MAC infection (resistant to mycobacterial chemotherapy), when administered alone to the patient [54]. 

#### 4.3.4. Shakanzoto

Shakanzoto (Zhi Gan Cao Tang) (9 herbs: including shakanzo *Glycyrrhiza uralensis*, *Rehmannia glutinosa*, *Ophiopogon japonicus*, *Cinnamomum cassia*, and *Daucus carota*) in combination with yokuinin (*Coix lacryma-jobi* var. frumentacea) exhibited excellent therapeutic effects on the clinical treatment of refractory pulmonary nontuberculous mycobacterial infection with persistent bloody sputum [56]. Shakanzoto is efficacious in treating arrhythmia, heart neurosis, and fatigue and yokuinin improves the water balance of body tissues. Kuwatani et al. reported that the oral administration of these two traditional herbal medicines reduced intractable bloody sputum within eight days of the initiation of their administration without subsequent recurrence of the symptoms [56]. 

#### 4.3.5. Chikuyosekkoto and Shigyakuto

The two traditional herbal medicines chikuyosekkoto (Chu Yeh Shih Kao Tong) and shigyakuto (Si Ni Tang) show favorable therapeutic efficacy against respiratory disease and chronic wasting disease, respectively. Shibahara et al. reported that chikuyosekkoto consisting of seven herbs, including *Phyllostachys*, *Pinellia*, *Daucus carota* and *Glycyrrhiza glabra*, and gypsumplaster, and shigyakuto (three herbs: *Glycyrrhiza glabra*, *Zingiber officinale*, and *Aconitum carmichaelii*) effectively improved the physical status, leading to acceleration of the negative conversion of MAC bacilli in sputum, when given to a patient with MAC infection in combination with antimicrobial chemotherapy using isoniazid, rifampin, and kanamycin [55]. 

These findings support the concept that some kinds of traditional herbal medicines exhibit mild but significant therapeutic effects against intractable mycobacteriosis due to non-tuberculous mycobacteria, especially when used in combination with antimycobacterial chemotherapeutics. In this context, Putri et al. reported an interesting finding concerning an Indonesian herb, *Phyllanthus niruri*, which has been used to boost the immune system [58]. They demonstrated that an aqueous extract from *P. niruri* induced the proliferation of human peripheral mononuclear cells, increased NO release, and promoted the phagocytic ability of macrophages.

## 5. Concluding Remarks

In the period before the establishment and application of antimycobacterial chemotherapy, symptomatic treatment of TB patients, including rest, nutrition, and sunlight exposure, was successful to some extent in arresting TB. This indicates that there is a significant capacity for self-cure in hosts with MTB infection, thereby suggesting the possible usefulness of host-directed therapy using immunoadjunctive drugs for the clinical management of TB and other intractable mycobacterioses, especially MAC infections. From this viewpoint, a number of HDTs, including herbal medicines/medicinal plants and their active chemical components, have been or are being developed for clinical use in the treatment of mycobacterial infections, as described above. 

In this paper, we reviewed the usefulness of herbal medicines/medicinal plants and their chemical components in the clinical treatment of intractable mycobacterial infections, especially in combination with antimicrobial chemotherapy, primarily based on basic and clinical reports. We feel that the investigations that have been or are being carried out in this specific field are insufficient. However, on the basis of recent findings with special reference to the immunological action of herbal medicines/medicinal plants and their chemical components, it can be concluded that certain herbal medicines/medicinal plants are promising as immunoadjuncts for the clinical control of mycobacteriosis. Notably, herbal medicines/medicinal plants can be divided into the following two types based on their immunological effects on hosts: (1) herbal medicines/medicinal plants exhibiting immuno-potentiative and/or proinflammatory effects, and (2) herbal medicines/medicinal plants exerting immunosuppressive and/or anti-inflammatory effects, depending upon individual agents and their active chemical components. Therefore, these profiles that are characteristic of individual herbal medicines/medicinal plants and their chemical components should always be taken under careful consideration when we use herbal medicines/medicinal plants as immunoadjunctive agents for the clinical treatment of intractable mycobacteriosis. 

As with a number of immuno-potentiating supplements and foods, which are widely used to up-regulate host immunity against microbial infections and tumors, herbal medicines/medicinal plants are thought to exert dual effects on the host immune system, as follows: (1) the up-regulation of innate immunity and acquired cellular immunity through the expansion of M1 macrophages and Th1 lymphocytes, and (2) immuno-regulation (suppression) leading to the reduction of tissue damage due to the excess manifestation of cellular immunity through the expansion of M2 macrophages and Th2 and Treg lymphocytes. Concerning these situations, it is desirable to make further advances in basic research with respect to the detailed immunological effects of various herbal medicines/medicinal plants and their chemical components in the near future. It will be necessary to elucidate the immunological properties of individual herbal medicines/medicinal plants, particularly in terms of their effects on Th1 and Th2 immuno-deviation and macrophage polarization into M1- and M2- type populations.

## Figures and Tables

**Table 1:** Immunological effects of active chemical components of herbal medicines.

Chemical Components	Herbs/Medicinal Plants	Remarks	References
1. Curcumin	*Curcuma longa*	Potentiation of anti-MTB activity of macrophages Blocking effect against M1 macrophage polarization due to inhibition of TLR2 activation of macrophages	[15,16]
2. Astragalus polysaccharide	*Astragalus membranaceus*	Potentiation of macrophage production of inflammatory cytokines (IL-6, IL-1β, TNF-α) Up-regulation of expansion of Th1 and Th17 cells Down-regulation of Treg and Th2 cells	[17,18,19]
3. Astrgaloside (polyphenol)	*Astragalus membranaceus*	Potentiation of macrophage production of inflammatory cytokines (IL-6, IL-1β, TNF-α)	[17]
4. Triptolide (diterpene triepoxide)	*Tripterygium regelii*	Supression of IL-2 production by T cells Reduction of macrophage production of IL-12 and IL-23	[20]
5. Berberine (isoquinoline alkaloid)	*Coptis japonica* *Phellodendron amurense*	Inhibition of the expansion of Th1 and Th17 cells but not Treg cells	[21]
6. Andrographolide (diterpenelactone)	*Andrographis paniculata*	Restoration of LPS-IFN-g-induced reduction of macrophage sensitivity to dexamesasone based on IL-27 generation	[22]
7. Piperlongumine (alkaloid)	*Piper longum*	Inhibition of dendritic cell maturation in response to LPS resulting in supression of inflammatory cytokines (IL-12, IL-6, TNF-α)	[23]
8. Osthole (Cumarin)	*Cnidium monnieri*	Suppression of Th2-cell-mediated asthma by inhibiting Th2-type cytokines (Il-4, IL-5, IL-13, but not IL-10) Blocking of dendritic cell maturation, resulting in lowered expression of inflammatory cytokines (IL-12, IL-6, TNF-α) but increased expression of IL-10	[24]
9. Root components	*Glycyrrhiza uralensis*	Reduction of matured IL-1β production by macrophages due to inhibition of caspase-1 expression	[25]
10. Fermented Gastrodiae	Gastrodiae rhizome (*Gastrodia elata*)	Suppression of macrophage production of reactive oxygen species, prostaglandin E_2_	[26]

**Table 2:** Clinical and experimental trials of Chinese herbal medicine (CHM) therapy against mycobacteriosis.

CHM/Study Design	Remarks	References
1. Meta-analysis (1823 MDR-TB patients)	CHM therapy combined with antimycobacterial chemotherapy was associated with superior treatment success and radiological improvement with low-level adverse effects.	[48]
2. Meta-analysis (3374 MDR-TB patients)	CHM therapy combined with antimycobacterial chemotherapy accelerated resorption of lung lesions and cavity closure.	[49]
3. Hochu-Ekki-To Randomized controlled trial (18 patients with MAC infection)	Hochu-Ekki-To therapy combined with antimycobacterial chemotherapy was effective in blocking the increase in bacterial loads in sputum and in improving radiological and nutritional conditions.	[50]
4. Ninjin-Youei-To (one patient with MAC infection)	Ninjin-Youei-To therapy combined with antimycobacterial chemotherapy was effective in accelerating negative conversion of MAC bacilli in sputum and in improving radiological features.	[51]
5. Ninjin-Youei-To (one patient with *M. fortuitum* infection)	Ninjin-Youei-To therapy alone caused negative conversion of bacterial loads in sputum.	[52]
6. Ninjin-Youei-To (one patient with *M. xenopi* infection)	Ninjin-Youei-To therapy alone was effective in increasing patient’s body weight and reducing sputum expectoration and cough.	[53]
7. Sainkan-To (one patient with MAC infection)	Sainkan-To therapy alone was efficacious in reducing anti-inflammatory reactions and anemia.	[54]
8. Chikuyo-Sekko-To and Shigyaku-To (one patient with MAC infection)	Therapy with Chikuyo-Sekko-To combined with Shigyaku-To accelerated negative conversion of MAC bacilli in sputum.	[55]
9. Shakanzo-To (one patient with MAC infection)	Shakanzo-To therapy alone was effective in diminishing bloody sputum without subsequent recurrence of the symptoms.	[56]
10. *Qi*-boosting and *Yin*-nourishing decoctions (one TB patient)	The two regimens in combination with anti-MTB chemotherapy were effective in accelerating the negative conversion of MTB bacilli in sputum and reducing lung lesions.	[57]
11. Niubeixiaohe (MTB-infected mice)	Niubeixiaohe treatment of mice with MTB infection decreased bacterial loads in the spleen accompanied by moderate improvement of histopathological features.	[43]
12. Mao-Bushi-Saishin-To (MAC-infected mice)	Mao-Bushi-Saishin-To therapy in combination rifamycin (rifalazil) of MAC-infected mice was mildly effective in reducing bacterial loads in the lungs. Mao-Bushi-Saishin-To increased anti-MAC activity of host macrophages.	[12]
13. Yokuinin (MAC-infected mice)	Yokuinin was mildly efficacious in inhibiting bacterial growth in murine macrophages when used in combination with rifamycin (rifalazil). Yokuinin did not potentiate therapeutic activity of rifalazil against MAC infection induced in mice.	[13]
14. Kinpouge/Kujin/Kagoso/Jinchouge (MTB-infected rats)	Water extract from the four herbs potentiated host resistance to MTB infection in rats.	[14]
